# *Phaeobacter inhibens* induces apoptosis-like programmed cell death in calcifying *Emiliania huxleyi*

**DOI:** 10.1038/s41598-018-36847-6

**Published:** 2019-03-21

**Authors:** Anna R. Bramucci, Rebecca J. Case

**Affiliations:** Department of Biological Sciences, University of Alberta, Edmonton, Alberta T6G 2E9 Canada

## Abstract

The model coccolithophore, *Emiliania huxleyi*, forms expansive blooms dominated by the calcifying cell type, which produce calcite scales called coccoliths. Blooms last several weeks, after which the calcified algal cells rapidly die, descending into the deep ocean. *E. huxleyi* bloom collapse is attributed to *E. huxleyi* viruses (EhVs) that infect and kill calcifying cells, while other *E. huxleyi* pathogens, such as bacteria belonging to the roseobacter clade, are overlooked. EhVs kill calcifying *E. huxleyi* by inducing production of bioactive viral-glycosphingolipids (vGSLs), which trigger algal programmed cell death (PCD). The roseobacter *Phaeobacter inhibens* was recently shown to interact with and kill the calcifying cell type of *E. huxleyi*, but the mechanism of algal death remains unelucidated. Here we demonstrate that *P. inhibens* kills calcifying *E. huxleyi* by inducing a highly specific type of PCD called apoptosis-like-PCD (AL-PCD). Host death can successfully be abolished in the presence of a pan-caspase inhibitor, which prevents the activation of caspase-like molecules. This finding differentiates *P. inhibens* and EhV pathogenesis of *E. huxleyi*, by demonstrating that bacterial-induced AL-PCD requires active caspase-like molecules, while the viral pathogen does not. This is the first demonstration of a bacterium inducing AL-PCD in an algal host as a killing mechanism.

## Introduction

Coccolithophores are well known for their precipitation of dissolved bicarbonate to produce characteristic ornate calcite disks or coccoliths^1^. They are globally important bloom-forming algae, frequently forming blooms that cover 100,000–250,000 km^2^ stretches of the upper ocean^2,3^. Coccolithophore blooms are primarily made up of the ubiquitous species complex of *Emiliania huxleyi*^4^, which is the most abundant and smallest coccolithophore in modern oceans^5^. These *E. huxleyi* populations are dominated by diploid coccolith-bearing cells, which grow rapidly, and often bloom to densities of over 10^5^ cells/mL in the upper ocean^6^. *E. huxleyi* bloom dynamics (peak density and subsequent crash) are determined by the rates of reproduction and death within the population. Phytoplankton death has several known causes including: algal senescence (aging), environmental stresses (e.g., nutrient deprivation, high irradiance, etc.), interactions with predators and pathogens, and programmed cell death (PCD)^7^. For example, the duration of coccolithophore blooms can be dramatically shortened by predation from microzooplankton^8^ and infection by viruses^9^. The latter can trigger premature collapse of *E. huxleyi* blooms by hijacking algal PCD pathways, inducing algal death^10–12^. Such a role for PCD in bloom collapse is not unique to *E. huxleyi* and has been observed in a number of other unicellular phytoplankton (prokaryotic and eukaryotic) such as cyanobacteria^13^, diatoms^14^, dinoflagellates^15^, and green algae^16^.

PCD is the potentially interruptible process through which an independent cell responds to internal or external signals by genetically initiating and then biochemically orchestrating its own deconstruction. Apoptotic-PCD (or apoptosis) was initially defined as having: (1) a strict reliance on the biochemical activity of highly specific proteases called caspases (i.e., cysteine aspartic proteases that cleave proteins after aspartic acid residues) and (2) conserved cellular morphologies during death (i.e., cell shrinkage, chromatin condensation, nuclear degradation, apoptotic bodies, etc.)^17,18^. Furthermore, the required dependence on caspase activity means that apoptosis can be abolished, or interrupted, by biochemically inhibiting caspases^18,19^. Caspases have not yet been identified in non-metazoans, which is why apoptosis was initially assumed to be a strictly metazoan process^20^. However, the identification of caspase-like peptide cleavage in plants and unicellular phytoplankton, which lack caspases^20^, led to the recognition of an alternate death process called: apoptosis-like-PCD (AL-PCD). AL-PCD is now used to describe PCD with characteristic apoptotic morphologies, but lacking the hallmark caspase activity^21^. Instead, AL-PCD can rely on either metacaspase or caspase-like protease activities. A few caspase-like proteases have been identified in plants, for example vacuolar processing enzyme (YVADase, caspase-1-like^22^), proteasomes (DEVDase, caspase-3-like^23^), and saspases (IETDase, caspase-8-like^24,25^), among others^26–28^. This diversity of enzymes with caspase-like activities explains why some ‘caspase-specific’ probes, such as those used in the current and previous studies^11,29^, are not exclusively specific to caspases.

Some bacteria have recently been shown to display pathogenicity toward the dominant calcifying *E. huxleyi*, while the non-calcifying diploid cells are resistant to bacterial pathogens^29,30^. These bacterial pathogens belong to the marine roseobacter clade, members of which can sense algal exudates and are closely associated with algal blooms in the open ocean^31–33^. *Phaeobacter inhibens* DSM 17395, for example, has been identified within blooming populations of *E. huxleyi*^34^ and was recently shown to be a pathogen of *E. huxleyi*^30^. This *P. inhibens* strain produces the *E. huxleyi* cell-cell signal, indole acidic acid (IAA)^32,35^, and several algaecidal bioactives such as roseochelins^36^ and roseobacticides^37^, which have been postulated to facilitate pathogenic interactions with calcifying *E. huxleyi*. However, the mechanism of algal death during this pathogenic interaction has not been elucidated.

Given that lytic *E. huxleyi* viruses (EhVs) produce bioactive viral glycosphingolipids (vGSLs) that trigger *E. huxleyi* PCD^11^ and/or autophagy pathways^12^, we hypothesized that bacterially induced AL-PCD might be the cause of algal death in this bacterial-algal interaction. To test this hypothesis, *E. huxleyi* was grown in co-culture with *P. inhibens* and monitored for previously identified PCD phenotypes associated with viral infection of *E. huxleyi* (i.e. generation of reactive oxygen species (ROS)^38,39^ and elevated caspase-like IETDase activities^11,29^). Not only were both of these phenotypes observed, but *P. inhibens* killing of *E. huxleyi* was also abolished by the addition of a pan-caspase inhibitor (z-VAD(OMe)-fmk). As AL-PCD requires active caspase-like molecules to propagate the death signal^21^, biochemical inhibition of algal death confirmed that the bacterium is inducing caspase-like dependent (or ‘z-VAD(OMe)-fmk’-inhibitable^18^) AL-PCD. This finding differentiates bacterial pathogenesis from viral infections^11,12^, by conclusively demonstrating a reliance on algal caspase-like molecules to propagate algal death. Algal cell death by AL-PCD was further confirmed by the observation of late-stage nuclear degradation and subsequent loss of cellular DNA^18,21^.

## Results

### *P. inhibens* enhanced reactive oxygen species (ROS) generation in *E. huxleyi*

**Figure 1:**
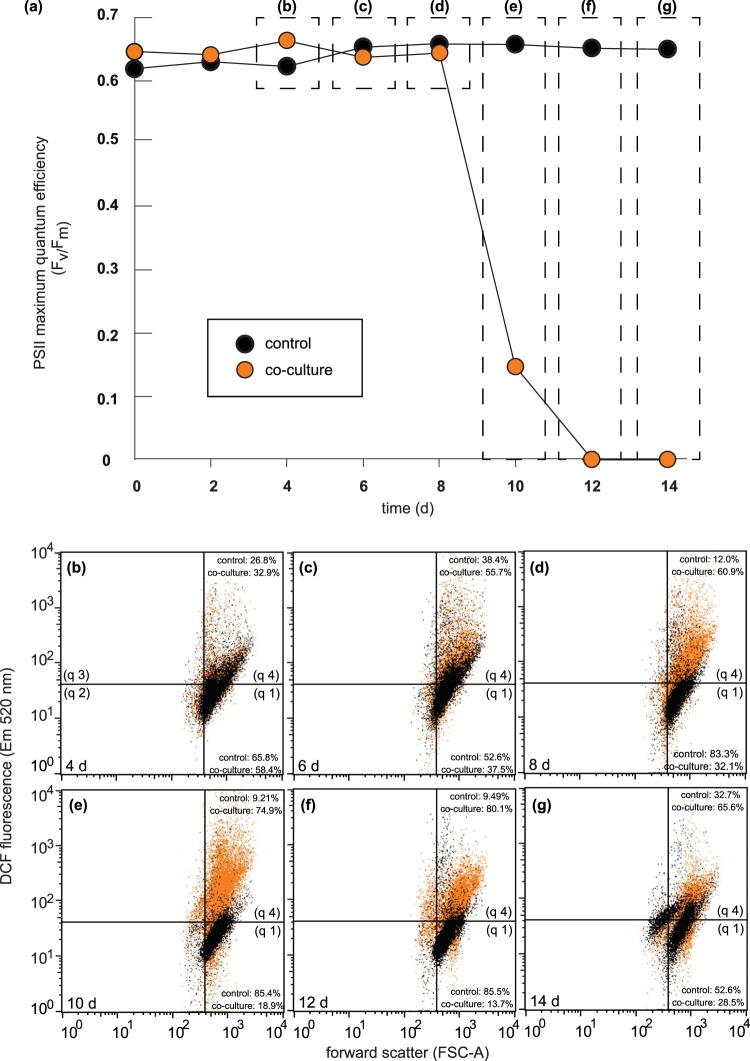
Reactive oxygen species (ROS) production stimulated in *Emiliania huxleyi* cells infected with *Phaeobacter inhibens*. Control cultures of *E. huxleyi* (black circles) and co-cultures of *E. huxleyi* grown with *P. inhibens* (orange circles) were assessed for (**a**) Photosystem II (PSII) maximum quantum efficiency levels and then immediately stained with general oxidative stress indicator (CM-H2DCFDA) to detect algal ROS. Samples were then immediately analysed using flow cytometry (fluorescence (excitation 488 nm, emission 520 nm) and cell size (FSC-A)). Data for control *E. huxleyi* cells (black dot plots) overlaid above co-culture of *E. huxleyi* and *P. inhibens* algal cells (orange dot plots): (**b**) 4 d, (**c**) 6 d, (**d**) 8 d, (**e**) 10 d, (**f**) 12 d, and (**g**) 14 d. The density of the dots is proportional to the density of the detections events. The vertical and horizontal quadrants are based on >95% of non-ROS-stained algal cells being in quadrant 1 (q1). Cells to the left of the vertical line show loss of algal cell size (FSC-A) and are therefore considered dead regardless of ROS staining (q2 and q3 total <10% of cells counted for both control and co-culture on all days). The proportions that control *E. huxleyi* cells present in either q1 (healthy) or q4 (elevated ROS) are as follows: 4 d (q1 65.8%, q4 26.8%); 6 d (q1 52.6%, q4 38.4%); 8 d (q1 83.3%, q4 12.0%); 10 d (q1 85.4%, q4 9.21%); 12 d (q1 85.5%, q4 9.49%); and 14 d (q1 52.6%, q4 32.7%). The proportions of *E. huxleyi* cells present in either q1 (healthy) or q4 (elevated ROS) after prolonged co-culture with *P. inhibens* are as follows: 4 d (q1 58.4%, q4 32.9%); 6 d (q1 37.5%, q4 55.7%); 8 d (q1 32.1%, q4 60.9%); 10 d (q1 18.9%, q4 74.9%); 12 d (q1 13.7%, q4 80.1%); and 14 d (q1 28.5%, q4 65.6%).

The interaction between the marine pathogen *P. inhibens* and the calcifying microalga *E. huxleyi* was examined by growing these two organisms alone and together in prolonged co-culture. During co-culture, the bacterial pathogen induced an accelerated loss of functional Photosystem System II (PSII), as measured by a dramatic decrease in PSII maximum quantum efficiency (Fig. 1a). This rapid decrease in PSII health is commonly used as an indicator of imminent death for photoautotrophs such as *E. huxleyi*^40^.

The dramatic loss of PSII health was also suggestive of ROS involvement in cellular destruction, which is a notable occurrence during EhV infection of *E. huxleyi*^38,39^. To monitor the proportion of algal cells with elevated intracellular ROS, the ROS indicator CM-H2DCFDA was added to algal cultures, where it was rapidly hydrolyzed by algal esterases, then oxidized by ROS in the cytoplasm, resulting in intracellular fluorescent dichlorofluorescein (DCF)^38,41^. Therefore, more ROS in an individual cell results in a higher DCF fluorescence. The shift in DCF fluorescence in each cell was measured using flow cytometry, and the cells were gated by the vertical and horizontal quadrants drawn in order to constrain >95% of control algal cells (without added ROS indicator CM-H2DCFDA) inside quadrant 1 (q1). The shift of algal cells from q1 (baseline DCF fluorescence) into q 4 (elevated DCF fluorescence) was used to determine the proportion of algal cells with elevated DCF fluorescence (i.e. elevated ROS within cells) (Fig. 1b–g). *E. huxleyi* control populations, which maintained a high PSII maximum quantum efficiency (>0.6) for the duration of the experiment (Fig. 1a), had a maximum of 38.4% of their cells showing elevated DCF fluorescence (Fig. 1b–g). The co-cultures, on the other hand, experienced a pronounced vertical shift in DCF fluorescence that coincided with the loss of photosynthetic efficiency of the algal population (10 d) (Fig. 1e). At that time, the proportion of algal cells in co-culture containing elevated DCF fluorescence increased to 74.9% of the population (q4), with only 18.9% of the remaining algae in co-culture having baseline levels of DCF fluorescence (q1) (Fig. 1a,e). The remaining 6.2% of the counted algal cells had shifted left into either q2 or q3, demonstrating a substantial loss of algal cell size (FSC) from healthy *E. huxleyi* (q1) and were therefore omitted from these proportions. By 12 d the proportion of algal cells in co-culture with healthy or baseline levels of ROS per cell had dropped to 13.7% of the population (Fig. 1e,f). Notably, the majority of algal cells in co-culture transitioned from normal ROS concentrations to elevated ROS concentrations (shifting from q1 to q4) between 8 and 10 d, at which time algal PSIIs were irreparably damaged (Fig. 1a).

### *P. inhibens* stimulated caspase-like activity in *E. huxleyi* as PSII efficiency declined

**Figure 2:**
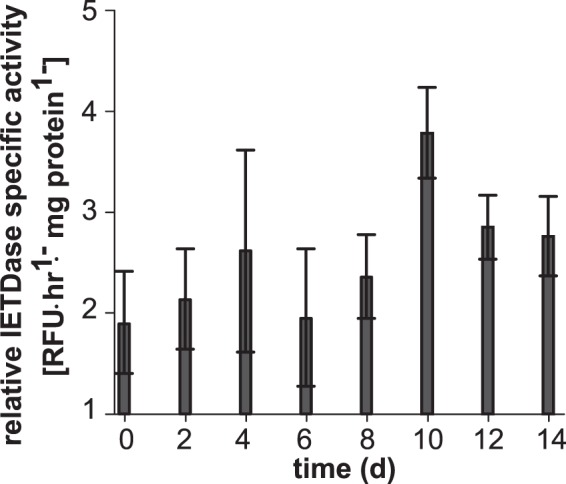
IETDase protease activity stimulated in *Emiliania huxleyi* infected with *Phaeobacter inhibens*. *In vitro* IETDase (Ile-Glu-Thr-Asp) activity in *E. huxleyi* control and co-cultures was measured using Caspase-8 Activity Kit. The ratio of IETD cleavage in *E. huxleyi* cells infected with the bacterial pathogen was normalized to IETD cleavage activity in uninfected control cells to depict relative increase in algal caspase-like enzymes capable of IETDase cleavage. Error bars (±SE) were calculated using the error-propagation equation.

To identify if pathogen-induced algal death might rely on caspase-like molecules to orchestrate AL-PCD, we next quantified caspase-like IETDase activities, which have previously been reported in *E. huxleyi*^10,29^. The relative increase in algal IETDase activity in bacterial-algal co-cultures compared to the controls was not significant between 0 and 8 d. However, as the PSII efficiency of the algal hosts crashed between 8 and 10 d (Fig. 1a), there was a simultaneous ~2-fold increase of IETDase activity in the co-culture relative to the axenic control (Fig. 2).

### Morphologies typical of AL-PCD were observed in *E. huxleyi* co-cultured with *P. inhibens*

**Figure 3:**
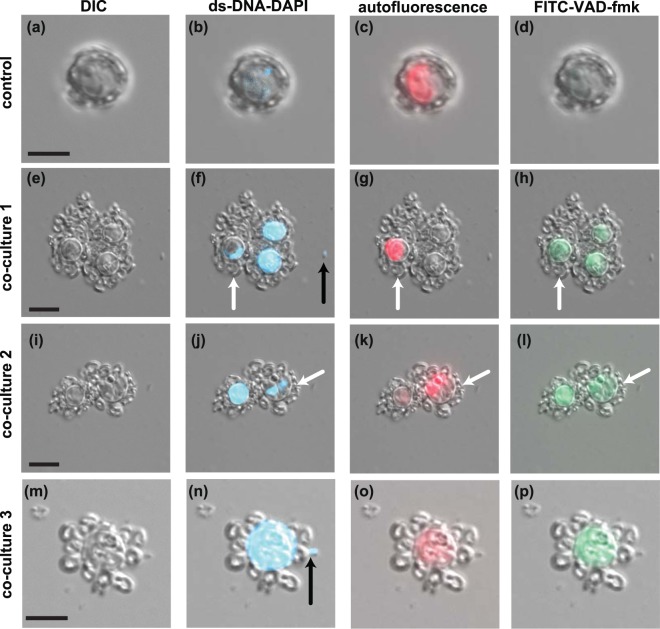
Morphological changes in *Emiliania huxleyi* cells infected with *Phaeobacter inhibens* characteristic of apoptotic like-programmed cell death (AL-PCD). DIC images of control *E. huxleyi* cells (10 d) (**a**) and three different co-cultures (**e**,**i**,**m**). Simultaneous images of DIC and three individually overlaid fluorescent channels: (1) DNA stained with DAPI (blue: excitation 350–400 nm; emission 417–477 nm) of control (**b**) and co-cultures (**f**,**j**,**n**). (2) chlorophyll autofluorescence (red: excitation 610–650 nm; emission 670–720 nm) in control (**c**) and co-cultures (**g**,**k**,**o**), (3) pan-caspase marker (v-VAD-FMK) highlighting active caspase-like proteases (green: excitation 450–490 nm; emission 515–586 nm) of algal control (**d**) and co-cultures (**h**,**l**,**p**). White arrows indicate healthy cells (indicated by chloroplast autofluorescence (red)). DAPI stained *P. inhibens* cells indicated by black arrows. Scale bar is 5 µm.

To further confirm *P. inhibens* infected *E. huxleyi* cells died via AL-PCD, widely reported AL-PCD morphologies (e.g., loss of nucleus membrane integrity^16,42^, loss of autofluorescence^11^, and active caspase-like molecules^10,11^) were observed for in co-cultures and controls. Throughout the experiment, control algal cells retained a defined nucleus (as seen by a compact and defined DAPI-stained region), chlorophyll autofluorescence, and had no visible staining with the pan-caspase marker FITC-VAD-fmk labeling (Fig. 3a–d). In contrast, *E. huxleyi* cells grown in co-culture with *P. inhibens*, began displaying apoptotic-like morphologies, such as nuclear degradation as early as 8 d (see: Supplemental Fig. S1). Nuclear degradation was followed by signs of active caspase-like molecules localized in or around the algal chloroplasts (see: Supplemental Fig. S2). By 10 d, most *E. huxleyi* cells grown with *P. inhibens* showed one or more AL-PCD morphologies (loss of intact nucleus, loss of autofluorescence, active caspase-like molecules) (Fig. 3). On 10 d, some cells were still autofluorescent, but FITC-VAD-fmk labeling was also observed in these cells (Fig. 3, white arrows), indicating that they had started AL-PCD but were not as far along in the process as algae lacking autofluorescence (Fig. 3g). As AL-PCD progressed, autofluorescence disappeared and a defined nucleus was no longer visible, instead DAPI stained DNA and FITC-VAD-fmk labeled active caspase-like molecules were visible throughout the cytoplasm (Fig. 3f,g,h).

### Pan-caspase inhibition restored *E. huxleyi’s* PSII efficiency and abolished *P. inhibens* induced AL-PCD

**Figure 4:**
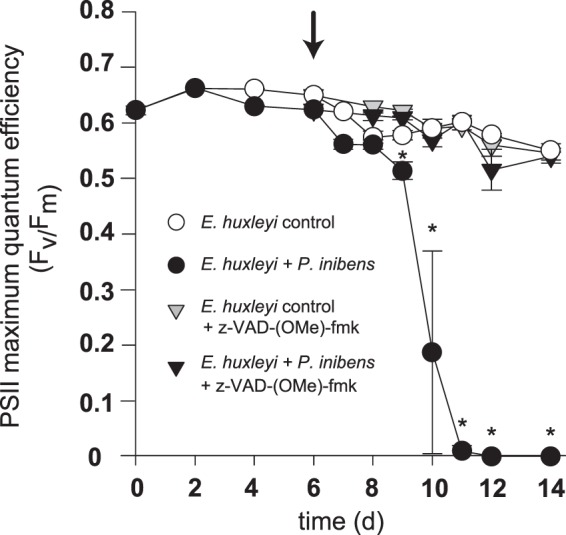
*Emiliania huxleyi’s* Photosystem II (PSII) maximum quantum efficiency declines when infected with *Phaeobacter inhibens*. PSII maximum quantum efficiency (Fv/Fm) of axenic *E. huxleyi* (white circles) and co-culture with *P. inhibens* (black circles). Cell permeable pan-caspase inhibitor added *in vivo* on 6 d at 20 µM z-VAD-(OMe)-fmk (black arrow), from that point on pan-caspase inhibited control *E. huxleyi* (light grey inverted triangles) and co-culture with *P. inhibens* (black inverted triangles) were also measured. Error bars = ±SE experimentally independent triplicate counts. An asterix (*) indicates that algal co-culture Fv/Fm is statistically different from the Fv/Fm of the non-inhibited algal control (Student’s T-test, p value < 0.001).

A hallmark of AL-PCD, differentiating it from autophagy, is that it can be abolished if caspase-like activities are inhibited^21^, while cells undergoing autophagy cannot be rescued by inhibiting caspase-like molecules^43^. To determine if this was the case for the pathogenic interaction between *P. inhibens* and *E. huxleyi*, the effect of a pan-caspase inhibitor on PSII efficiency and cell death was assessed. Additionally, to narrow the timeline of *E. huxleyi* death, this second experiment employed daily sampling once algal senescence was observed, enabling us to pinpoint the 24 hr period of algal death. PSII efficiency was monitored by again tracking *E. huxleyi’s* maximum quantum efficiency (F_v_*/*F_m_) throughout the experiment (Fig. 4). While grown in monoculture, *E. huxleyi* displayed high PSII efficiency throughout the 14 d experiment (Fig. 4). During the first six days of growth with *P. inhibens, E. huxleyi* PSII efficiency was slightly lower, although not statistically different from that in algal monocultures (Fig. 4). As the co-culture progressed however, algal PSIIs showed signs of irreparable damage (10 d) and PSII efficiency declined dramatically (Fig. 4). However, when the pan-caspase inhibitor (z-VAD-(OMe)-fmk) was added to co-cultures on 6 d, *P. inhibens* induced loss of PSII efficiency in the algal host was completely abolished (Fig. 4). The pan-caspase inhibitor added to control algal cultures resulted in no statistically significant effect on algal PSII efficiency (Fig. 4).

**Figure 5:**
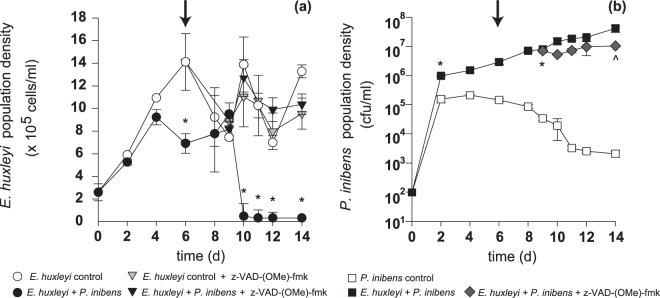
Bacterial and algal population dynamics. (**a**) *Emiliania huxleyi* cell density of control (white circles), co-culture with *Phaeobacter inhibens* (back circles), pan-caspase inhibited *E. huxleyi* (light grey inverted triangles) and pan-caspase inhibited *E. huxleyi* co-culture with *P. inhibens* (black inverted triangles). Cell permeable pan-caspase inhibitor (z-VAD-(OMe)-fmk) was added at 6 d (black arrow). An asterix (*) indicates that *E. huxleyi’s* cell density (cells/mL) in the control and co-culture treatments are statistically different. (**b**) *P. inhibens* cell density (CFU/mL) counts in control (white squares) and co-culture (black squares), and pan-caspase inhibited co-culture (grey diamonds) treatments. Error bars = ±SE experimentally independent triplicate counts. An asterix (*) indicates that *P. inhibens* cell density in control, co-cultured and caspase -inhibited co-culture and statistically different and (^) indicates that the pan-caspase inhibited co-culture is statistically different from the co-culture (Student’s T-test, p value < 0.001). Error bars = ±SE experimentally independent triplicate counts.

To test the effect of the pan-caspase inhibitor on algal cell death, *E. huxleyi* cell density in control monocultures and co-cultures with *P. inhibens* were also monitored. In monocultures, algal cell density initially increased exponentially (0–6 d), followed by a period of gradual algal senescence, typified by a gradual decline in algal cell density for a short period after reaching its maximum cell density (6–9 d) (Fig. 5a). The algal-bacterial co-culture displayed a lower maximum cell density at stationary phase, which remained stable until 9 d. As the algal controls and co-cultures entered late-senescence (9 d), the co-cultures underwent a rapid decline in cell density (i.e. death), irreversibly losing over 90% of the algal cells from the population in <24 hr (9–10 d) (Fig. 5a). The timing of this coordinated cell death event occurred in conjunction with the loss of PSII function (Fig. 4). While the *E. huxleyi* control cultures began to oscillate in stationary phase, rapidly rebounding after a limited period of decline, the algal cells grown in co-culture continued to irreversibly decline (Fig. 5a). Outer membrane integrity was maintained in the few algal cells remaining in the co-culture, until after the loss of all functional PSII systems (11–14 d) (Fig. 4). Again, pan-caspase inhibition at 6 d abolished *E. huxleyi* cell losses from the co-cultures and did not alter algal control population dynamics (10–14 d) (Fig. 5a).

### *P. inhibens* benefits from killing *E. huxleyi*

Bacterial monocultures grown in L1-Si medium (which is an enriched seawater medium made from filter-sterilized seawater that is autoclaved in small batches to minimize loss of organic nutrients) rapidly reached 10^5^ CFU/mL and then gradually declined until the end of the experiment (10^3^ CFU/mL) (Fig. 5b). As early as 2 d, *P. inhibens* grown in co-culture with *E. huxleyi* had a statistically significant increased bacterial density compared to the bacterium grown alone (Fig. 5b). As *E. huxleyi* cells died (9–10 d) (Fig. 5a), *P. inhibens* cell density almost doubled (Fig. 5b). This second exponential growth phase after reaching stationary phase coincides with the massive death event of the algal hosts (10 d) (Fig. 5a). The pan-caspase inhibitor, added at 6 d, was not detrimental to the pathogen, which displayed a similar population size in the uninhibited co-culture until 8 d. Interestingly, the bacterial population in the pan-caspase inhibited co-culture, where no algal death occurred (Fig. 5a), remained in stationary phase for the rest of the experiment (Fig. 5b).

### Algal cells in late-stage AL-PCD experience DNA degradation and loss of cellular integrity

**Figure 6:**
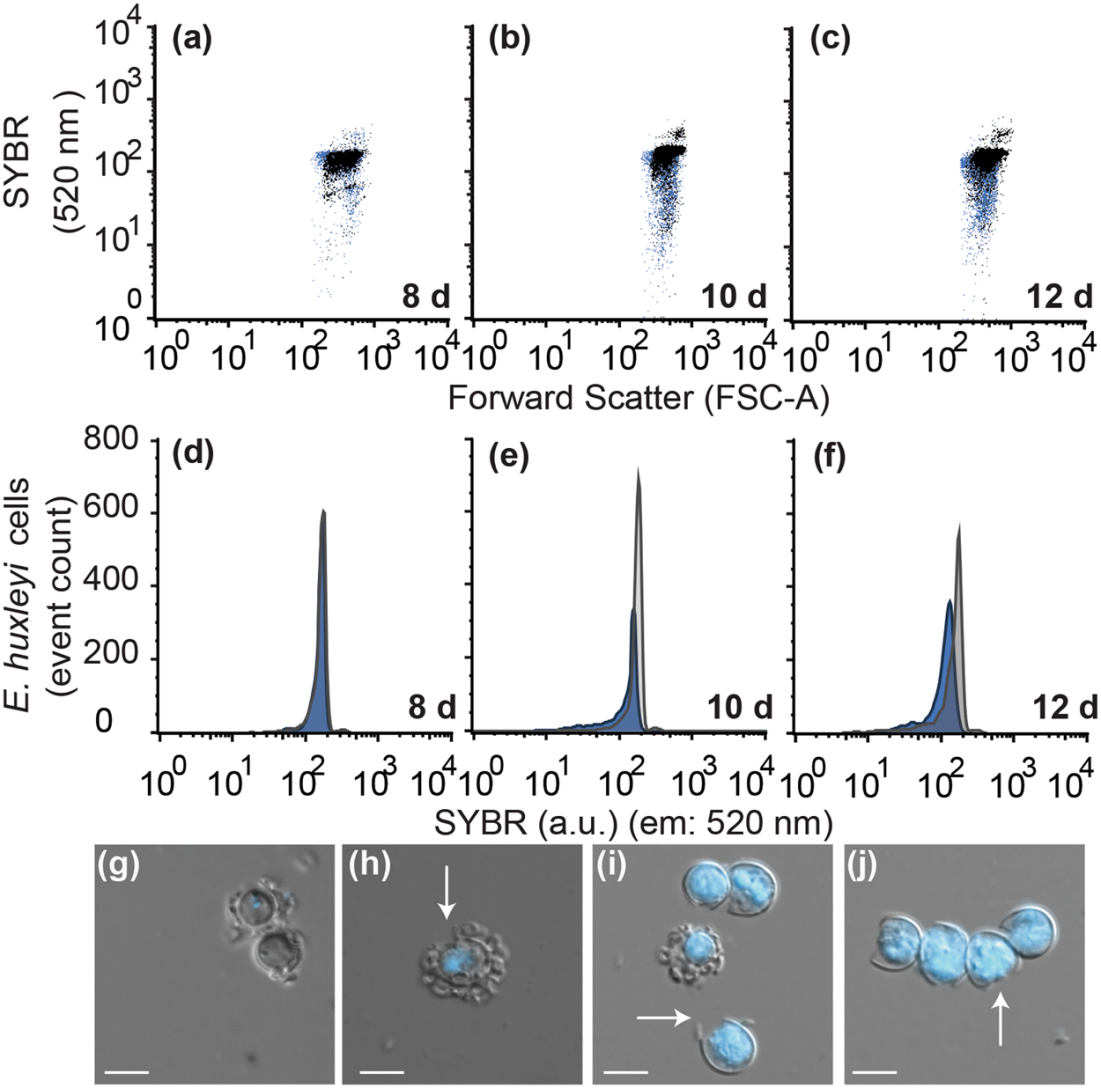
*Emiliania huxleyi*’s DNA is degraded and nucleus lost when infected with *Phaeobacter inhibens*. Flow cytometry of *E. huxleyi* cells co-cultured with *P. inhibens* and grown alone (control) stained with DNA specific SYBR green stain (520 nm) was performed to analyse the relative cellular DNA content. *E. huxleyi* (control) are represented by black dot plots, while co-cultured *E. huxleyi* is represented in blue dot plots: (**a**) 8 d, (**b**) 10 d and (**c**) 12 d. The density of the dots is proportional to the density of the detections events. Histograms of cell number (y-axis) and SYBR green stained cells are plotted on a logarithmic scale (x-axis) for co-cultures (blue) compared to controls (grey): (**d**) 8 d, (**e**) 10 d and (**f**) 12 d. Images show 12 d control (**g**) and co-culture (**h**–**j**), with DIC overlaid on DAPI stained DNA (blue: excitation 350–400 nm; emission 417–477 nm).

DNA cleavage and degradation is a common, though not required, feature found in metazoans undergoing apoptosis^18^ and in plants undergoing AL-PCD^21,28^. To determine if this phenotype was present, the DNA content of each algal cell was monitored using flow cytometry. On 8 d, the cellular DNA content of *E. huxleyi* populations in the controls and co-cultures were similar (Fig. 6a,d). The initial decline in *E. huxleyi* cell density in co-cultures occurred when the majority of algal cells died at 10 d (Figs 5a and 6e). After this, the remaining algal cells with intact cellular membranes in co-culture display decreasing DNA concentrations per algal cells compared to the control. This loss of DNA is depicted from flow cytometry analysis as the loss of DNA stained SYBR fluorescence from algal cells in co-culture (smeared population shifting vertically down), compared to control algae in flow cytometry plots in which cells all have a similar SYBR fluorescence and therefore DNA content (10–12 d) (Fig. 6b,c) and in histograms their shift left in SYBR fluorescence signifying a lower DNA content per cell on the logarithmic scale (Fig. 6e,f). As *E. huxleyi* cells began lose DNA (12 d), the algal cells from co-cultures lacked an identifiable nucleus (Fig. 6h–j). At this time, calcified and decalcified diploid cells (which have shed their coccoliths) both began showing signs of visibly damaged cellular membranes (Fig. 6h–j, white arrows).

## Discussion

This research establishes that the bacterial pathogen, *P. inhibens*, dynamically interacts with its calcifying microalgal host. During the interaction, the *E. huxleyi* cells display several indicators of imminent death by AL-PCD, such as elevated cellular ROS concentration, loss of PSII function^44^, and caspase-like activities^11,29^. Additionally, the algal host experiences several morphological changes previously associated with algal cells undergoing AL-PCD, such as chlorophyll degradation (loss of autofluorescence^11^), nuclear degradation (progressing to extra-nuclear DNA throughout the cell^45^), and DNA degradation (loss of DNA content per cell^29^). These morphologies are suggestive that the host is undergoing caspase-like directed AL-PCD, as they provide evidence that caspase-like molecules are cleaving integral cellular proteins that are required for cellular function, such as active photosynthesis or a functioning nuclear membrane. However, it is unadvisable to rely solely on these phenotypes as confirmation of death through the AL-PCD pathway, as the identities of the genes responsible for these caspase-like activities in phytoplankton are still largely unknown^7^.

The caspase-like activities direct the degradation of cellular proteins and the consequential loss of critical functions rapidly (<24 hr) resulting in algal death, conservatively defined as the fragmentation of the nucleus and/or cell membrane^18^. However, fitting these events to a timeline of algal death is challenging, as PCD is a cellular phenomenon, not a population-wide phenomenon^18,21^. This explains why a portion of the cells undergoing AL-PCD appear healthier than other cells even on the same day (Fig. 3, white arrows). By definition PCD occurs in each individual cell without propagating to nearby cells^18,21^, which means that the portion of cells undergoing AL-PCD is staggered. This can be observed in the ROS and SYBR fluorescently labeled populations in which differences between individual cells within the population produce a smear when compared to the tight population of uninfected control (Figs 1 and 6). This staggered onset of AL-PCD does not explain why a small portion of the algal cells in co-culture die between 4 and 6 d, before the pan-caspase inhibitor was added to cultures (Fig. 5a). Given that *P. inhibens* produces several algaecides that might be differentially regulated (e.g. roseochelins and roseobacticides^36,37^), it is possible that the two distinct types of cell death are caused by different effectors. Alternatively, a host senescent signal might be required for the production of the bacterial effector that triggers algal AL-PCD and induces the rapid decline of algal population on 10 d (Fig. 5a). Roseobacticides have indeed been shown to be produced only in the presence of *p*-coumaric acid, thought to be released by senescent *E. huxleyi* cells^37^. So, the cell losses occurring between 4 and 6 d might be caused by a different bacterial effector which does not respond to senescence signals and does not directly affect the photosynthetic apparatus. This earlier decline in algal cell density is not linked to a statistically significant decrease in photosynthetic health of the algal cells (Fig. 4), which is suggestive that these cells may not be dying from AL-PCD.

One overlap between metazoan apoptotic morphologies and those identified in algal AL-PCD is the loss of the nuclear membrane and subsequent loss of cellular DNA, which was first noted in the unicellular chlorophytes *Dunaliella tertiolecta* and *Chlamydomonas reinhardtii*^16,42,45^. During *C. reinhardtii* AL-PCD, nuclear degradation, or loss of an intact nuclear membrane, quickly progresses to extra-nuclear DNA throughout the whole cell^45^, similar of what we observed for *E. huxleyi* infected with *P. inhibens* (10 d) (Fig. 3). Additionally, rapid degradation and recycling of cellular DNA can quickly result in a decline in DNA content per cell^29,46,47^, due to the degradation of extra-nuclear DNA (Fig. 6). However, not all classical apoptosis morphologies are observed during AL-PCD^18,21^. For example, late-stage cellular membrane blebbing into apoptotic bodies is considered one of the hallmarks of apoptosis^48^, but is not a common feature of plants undergoing AL-PCD^49,50^. The lack of cellular membrane blebbing in calcifying *E. huxleyi* might be due to the presence of several layers of rigid coccoliths surrounding the cell. A similar phenomenon was reported during late-stage *D. tertiolecta* AL-PCD^16,51^, which could be attributed to *D. tertiolecta*’s rigid outer glycocalyx surface coat. Alternatively, it is possible that membrane blebbing is not a feature of AL-PCD^7^, differentiating it from metazoan apoptosis. In the experiments presented in this work the calcifying cultures used in both controls and co-cultures retained calcification throughout the experiments (Fig. 3). However, as the *E. huxleyi* cells grown in co-culture entered late-stage AL-PCD they did eventually show signs of shedding coccoliths (12 d), which coincided with signs of broken cellular membranes (Fig. 6h–j). Similar reports of coccolith shedding have been previously noted in virally infected *E. huxleyi* cultures^52,53^ and in naturally occurring blooms of *E. huxleyi*, where free floating coccoliths scatter light in the open ocean^6^.

Phytoplankton PCD encompasses all known PCD pathways that have been identified in unicellular phytoplankton^7^, including: AL-PCD (caspase-like dependent)^21^, paraptosis (non-apoptotic PCD)^54^, ferraptosis (iron-dependent)^55^, and autophagy (ATG gene dependent)^56^. PCD has been implicated as a critical cell death pathway in a variety of unicellular phytoplankton and its activation mechanisms are diverse. For instance, the diatom *Thalassiosira pseudonana* undergoes PCD in response to iron starvation^57^, while the unicellular chlorophyte *Dunaliella tertiolecta* responds to prolonged darkness^16^. The only known inducers of PCD in *E. huxleyi* before this study were lytic EhVs^58^. In this process, the virus induce algal synthesis of viral glycosphingolipids (vGSLs) that accumulate and subsequently trigger host-directed PCD^11,58,59^. Recently, EhVs were also shown to induce autophagy in a representative non-calcifying *E. huxleyi* strain^12^, demonstrating two potentially different mechanisms for EhVs to kill *E. huxleyi*. Despite the similarities between the algal death process during infection by EhVs and *P. inhibens*, there are some notable differences. For instance, the timing of viral-induced collapse of *E. huxleyi* cultures tends to occur at a steady rate over two to three days^11,12^, whereas *P. inhibens* induces a rapid *E. huxleyi* death event in the 24 hr period following 9 d (Figs 4 and 5a). Additionally, while biochemical inhibition abolishes bacterial induction of AL-PCD, viral killing of *E. huxleyi* is delayed, rather than abolished, by the same pan-caspase inhibitor^11^. This suggests viral lysis is not strictly dependent on caspase-like molecules as is *P. inhibens* pathogenesis of *E. huxleyi*. Viruses are known to delay eukaryotic apoptosis, rerouting the cell to the slower process of autophagy^60,61^, which could be occurring during EhV infection of *E. huxleyi*. Autophagy is a major degradation and recycling system requiring the packaging of cellular constituents into autophagy lysosome vesicles that are degraded intracellularly^62^, which would benefit viruses by indirectly supporting the production and release of virions^61^.

*E. huxleyi* senescence (characterised by a slower decline in cell density after reaching maximum cell density^63^) coincides with a slight decline in PSII efficiency, suggestive that photoinhibition of *E. huxleyi* (i.e. the control) could be involved in senescence. *E. huxleyi* has an ability to recover from photoinhibition, having an extensive repertoire of PSII repair proteins that repair PSII damage^4^. Importantly, *E. huxleyi* senescence is not reduced or delayed by pan-caspase inhibition in either the control or inhibited co-culture (Fig. 5a). This demonstrates that algal senescence is likely not regulated by AL-PCD and is not dependent on any ‘z-VAD(OMe)-fmk’-inhibitable caspase-like molecules, such as those critical to the pathogen induced algal death (Fig. 5). This finding suggests *E. huxleyi* senescence is regulated by an alternative death pathway, such as autophagy^18^. Similar autophagy-like processes have been identified in several plants and other algal species^64^, but the role of autophagy in *E. huxleyi* senescence has not yet been described.

It is not presently known how *P. inhibens* induces *E. huxleyi’s* AL-PCD, however previous work has shown that the potent bacterial algaecides called roseobacticides do not kill the strain of *E. huxleyi* used in this study (CCMP3266), and therefore we can conclude that roseobacticides do not activate AL-PCD in *E. huxleyi* CCMP3266^30^. In order to identify a potential bacterial virulence factor that induces algal AL-PCD, we are presently screening a transposon library of avirulent *P. inhibens* mutants in co-culture with CCMP3266 to identify virulence factors and/or effector molecules.

Bacterial pathogens have a plethora of strategies for infecting and killing host cells, some of which enable them to directly control the fate of a host cell by hijacking the PCD pathway. For example, *Shigella flexneri* and *Salmonella typhimurium* utilize a T3SS to inject bacterial proteins into host macrophages, directly activating specific caspases (i.e., caspase-1) and inducing apoptosis^65,66^. A similar mechanism is used by various *Yersinia* species, which inject several proteins in their host using T3SS that can both activate host caspases and inhibit host survival pathways, resulting in apoptosis^67^. Similar mechanisms are used by plant pathogens to induce AL-PCD of plant hosts. For instance, *Erwinia amylovora* injects T3SS effectors that either directly activate plant VPEs (likely stored in vacuoles, vesicles, or chloroplasts^68^) or induce ROS in the cell, both of which can lead to AL-PCD of apple leaf cells^69^. There has so far been no example of a bacterial pathogen inducing PCD in a unicellular alga as a killing mechanism. However, as the previously identified inducer of *E. huxleyi* PCD is a small bioactive (vGSL)^58^, it might stand to reason that the *P. inhibens* induce the same PCD machinery in its host by injecting a bacterially produced virulence factor or bioactive effector molecule.

## Conclusion

*E. huxleyi* bloom collapse is frequently attributed to various intracellular coccolithoviruses, which infect blooming populations of *E. huxleyi* in the open ocean^52^. However, the associated bacterial consortia are frequently overlooked, even though marine pathogens, such as those from the roseobacter clade, can track algal populations and sense and respond to algal exudates^32,34,70–73^. As ocean temperatures continue to rise, viral pathogens might become less of a threat to the longevity of *E. huxleyi* blooms, as viral resistance is enhanced with increasing temperatures^74^. Conversely, roseobacter pathogens were recently found to have enhanced pathogenesis of *E. huxleyi* at higher temperatures^29^. Here we demonstrate that bacterial pathogens can also cause rapid induction of AL-PCD in calcifying *E. huxleyi* cultures. This results in a rapid algal death event where over 90% of the algal population is lost from the co-culture in <24 hr, allowing the pathogen to rapidly double its population density. This increase in pathogen population density can be explained by the release of algal metabolites during pathogen-induced AL-PCD. This supports previous suggestions^13^ that pathogen-induced rapid algal death may change the way algal metabolites flow through the marine ecosystem, possibly accelerating the transfer of DOM to heterotrophic microbes.

## Methods

### Bacterial and algal strains

The axenic diploid coccolith-bearing *Emiliania huxleyi* CCMP3266, hereafter called *E. huxleyi*, was obtained from the Provasoli-Guillard National Centre for Marine Algae and Microbiota (NCMA). *E. huxleyi*, were maintained in F/2 medium (as it is at NCMA) and then transferred to L1-Si seawater medium and grown statically at 18 °C in a diurnal incubator (8:16 hr dark-light cycle) with 41.51 +/− 11.15 μmol/m^2^/s of light during the light period for 5 d prior to experiments to reach a density of 10^5^ cells/mL (early-log)^75^. Seawater for F/2 and L1-Si media was collected from the Bamfield Marine Sciences Center, BC, Canada and then filter-sterilized through a 0.22 µM filter and autoclaved in small batches to reduce autoclaving time to preserve organic nutrients before making algal media. Algal cultures and controls were routinely checked for bacterial contamination via microscopy and plated onto half-concentration marine agar (½MA) (18.7 g Difco Marine Broth 2216, 9 g NaCl, supplemented with 15 g Difco agar in 1 L of distilled water) incubated at 30 °C for 48–72 hr.

*Phaeobacter inhibens* DSM 17395, hereafter called *P. inhibens*^76,77^ was grown on ½MA plates or half-concentration marine broth (½MB) (18.7 g Difco Marine Broth 2216, 9 g NaCl, supplemented in 1 L of distilled water) at 30 °C. Prior to experimentation, freshly grown *P. inhibens* colonies were transferred to 25 mL ½MB, grown to early stationary phase at 18 °C in a shaking incubator (160 rpm, 30 hr), and then re-cultured under the same conditions. To ensure minimal carryover of nutrients from the bacterial inoculum to the co-culture with algae, the bacterial cells were washed twice via centrifugation and resuspension in sterile algal media prior to serially diluting the bacterial cells in algal media to the desired 0 d concentration (~10^2^ CFU/mL)^30,75^.

### Algal and bacterial co-cultivation

The dynamic interaction between the marine pathogen *P. inhibens* and the calcifying microalga *E. huxleyi* was examined by growing these two organisms alone and together in prolonged co-culture in two identical experiments, each of which had three independent replicates. On 0 d, algal and bacterial monocultures were mixed 1:1 with each other to obtain an evenly mixed co-culture, and each monoculture was then mixed 1:1 with sterile algal media to obtain two separate monocultures (to obtain a dilution ratio identical to that of the co-cultures). *E. huxleyi* and *P. inhibens* were co-cultured with initial cell concentrations of 2.6 × 10^5^ cells/mL and 1.2 × 10^2^ CFU/mL, respectively. Next, 1 mL aliquots of control algal and bacterial monocultures, as well as experimental co-cultures were gently pipetted into triplicate microtiter plate wells, as previously described^30,75^. The resulting microtiter plates were incubated statically at 18 °C in a diurnal incubator and sacrificial sampling was used throughout the experiment.

### Pulse-amplitude-modulated (PAM) fluorometry

Algal photosynthetic health was determined using a PAM fluorometer (WATER-PAM, Heinz, Walz). This method, explained in detail elsewhere^40,75,78,79^, is commonly used to non-invasively measure the overall health and efficiency of algal Photosystem II (PSII)^78^. In brief, WATER-PAM is used to determine minimal algal fluorescence of dark-adapted cells (F_0_), the maximum fluorescence (F_m_), and the ratio of these two values, which determines the PSII maximum quantum efficiency (F_v_/F_m_), F_v_/F_m_ = (F_m_ − F_0_)/F_m_^40,78,80^. All PAM measurements were taken at the mid-point of the dark cycle and diluted in L1-Si medium to within the detection range of the PAM fluorometer, as previously described^75^.

### Reactive oxygen species (ROS)

Intracellular ROS were measured for *E. huxleyi* cells using the membrane permeable 5-(and-6)-chloromethyl-2′,7′-dichlorodihydrofluorescein diacetate (CM-H2DCFDA; Invitrogen) fluorescent ROS probe (Molecular Probes Inc.). The probe diffuses passively into live cells, is hydrolyzed by intracellular esterases to 2′,7′-di-chlorohydrofluorescin (DCFDA), and remains trapped within the cell. The DCFDA is rapidly oxidized to the fluorescent compound dichlorofluorescein (DCF) and DCF concentration per algal cell is plotted using flow cytometry (detailed below). First, fresh 1 mM CM-H2DCFDA stock solution was made in dimethyl sulfoxide (DMSO) before use. Cells were incubated with final concentration 5 mM CM-H2DCFDA in the dark^38,81^. Flow cytometry was performed using a FACSCalibur with a 488 nm excitation laser (Becton Dickinson). Samples were run continually for 30 s using particle size based on side scatter (SSC) for detection and then analysed based on DCF fluorescence (green: emission 520 nm) and cell size (FSC-A).

Flow cytometry data were processed using FlowJo 9.2. Populations were gated into quadrants (q) based on the forward scatter and DCF fluorescence of a non-CM-H2DCFDA-stained 5 d old axenic culture. Using this method, >95% of non-stained algal cells were constrained within q 1, which can be defined as an algal cell without DCF fluorescence. Then q 2 and q 3 contained cells with decreased size (proportion <10% of the total), and q 4 is defined as algal cells of the expected cell size, but with elevated DCF staining (or ROS) per algal cell.

### Quantification of IETDase activity

*In vitro* IETDase (Ile-Glu-Thr-Asp) activity in *E. huxleyi* cells was measured as previously described^11^. Briefly, triplicate 950 uL aliquots from control and co-culture wells were pelleted by centrifugation (14,000 × g, 4 °C, 10 min), immediately flash-frozen in liquid nitrogen, and stored at −80 °C until processed. Cells were resuspended and a subsample was used for protein extraction (extraction buffer: 20 mM Tris pH 8.0, 100 mM NaCl, 1 mM MgCl_2_) and Bradford protein assay (BioRad). The remainder of the sample was centrifuged (16,000 × g; room temperature; 2 min), resuspended in caspase activity buffer, and sonicated, before pelleting of cellular debris (16,000 × g; room temperature; 2 min). The supernatant was then incubated with IETD-AFC (Ile-Glu-Thr-Asp-7-amino-4-trifluoromethylcoumarin) according to the manufactures instructions (Caspase-8 Activity Kit, EMB Millipore). Extracts were incubated for 4 hr at 25 °C and fluorescence (excitation 400 nm, emission 505 nm) was measured using a Synergy H1 microplate reader (BioTek). *In vitro* caspase activity was successfully abolished (>95%) with the irreversible caspase inhibitor z-VAD-fmk at a 20 µM final concentration (z-Val-Ala-Asp-fluoromethyl-ketone; Calbiochem), as previously described^11,29^. IETDase activities of the cellular extracts were normalized to protein content (RFU ∙ hr^1−^ ∙mg protein^1−^) for each extract prior to calculating the ratio of IETDase activities in algal co-cultures compared to IETDase activities in algal controls.

### Inhibition of caspase-like protease activity

*E. huxleyi* was grown alone and in co-culture with *P. inhibens* for 6 d in a microtiter plate; then all wells were mixed with a wide mouth pipette to suspend algal cells. Active caspase-like molecules were inhibited in the algal controls or co-cultures using the cell permeable and irreversible pan-caspase inhibitor: z-VAD(OMe)-fmk (z-Val-Ala-Asp-(OMe)-fluoromethyl-ketone). The inhibitor was added to each replicate *in vivo* on 6 d at a final concentration of 20µM v-VAD(OMe)-fmk.

### Algal and bacterial enumeration

Aliquots from control *E. huxleyi* and co-cultured with *P. inhibens* were fixed and run for flow cytometry analyses, as previously described^30^. Briefly, each time point was sacrificially sampled and aliquoted into subsamples for various analyses. Aliquots for flow cytometry were fixed 0.15% glutaraldehyde (Sigma-Aldrich), incubated for 10 min in the dark, then flash-frozen in liquid nitrogen and stored at –80 °C until processed using a FACSCalibur (Becton Dickinson), equipped with a 488 nm excitation laser. Samples were stained with SYBR-I (Life Technologies) (emission = 520 nm) to enumerate algal cells. Data were processed using FlowJo v9.2 (Tree Star Inc.).

*P. inhibens* population density from the control (without *E. huxleyi*) and co-cultivation experiments were enumerated by performing by colony forming unit (CFU) on ½MA (30 °C for 48 hr).

### Microscopy

Aliquots were collected during the experiment for brightfield, differential phase contrast (DIC), and epifluorescence microscopy. Brightfield images were obtained using Zeiss Axio Scope.A1, equipped with an optronics digital camera and PictureFrame Software Ver 2.3. DIC and epifluorescence images were obtained using a Zeiss Axio Imager.M2 microscope, equipped with a monochrome camera (AxioCam 506 mono). Active algal caspase-like proteases were visualized using epifluorescence microscopy after *in vivo* staining of cells with the cell permeable, irreversibly binding, pan-caspase fluorogenic probe, for detection of activated caspases in living cells (FITC-VAD-fmk, Millipore). The pan-caspase marker binds to active caspase and caspase-like proteases^82^, having both the characteristic cysteine—histidine dyad and an available Val-Ala-Asp (VAD) binding site. This method has been used effectively to label activated caspase-like molecules in *E. huxleyi*^11,29^. Epifluorescence microscopy was also used to image chlorophyll auto-florescence and to visualize changes in the chromatin using the DNA stain DAPI (4′; 6-Diamidino-2-phenylindole dihydrochloride) (Ex = 364 nm, Em = 454 nm) (Life Technologies).

Aliquots of unfixed *E. huxleyi* cells from the control and co-cultures were stained with pan-caspase marker (FITC-VAD-fmk) and DAPI according to manufacturer’s instructions (30 °C, 20 min), then immediately pelleted by centrifugation (5,000 × g, room temperature, 2 min). Cells were gently washed twice in filter sterile L1-Si medium and analysed immediately on the epifluorescence microscope. Images were acquired simultaneously for differential interference contrast (DIC) and three fluorescent channels, which were subsequently overlaid with the same corresponding DIC using Zen 2 Blue Edition software. DIC was individually overlaid each of the following three fluorescent channels: 1) chlorophyll auto-fluorescence (red: excitation 610–650 nm; emission 670–720 nm), 2) localization of active caspase-like proteases using specific pan-caspase marker: FITC-VAD-fmk (green: excitation 450–490 nm; emission 515–586 nm, green), 3) localization of the nucleus based on dsDNA-DAPI complex fluorescence (blue: excitation 350–400 nm; emission 417–477 nm).

## Electronic supplementary material

Supplemental File

## Data Availability

The datasets generated during and/or analysed during the current study are available from the corresponding author on reasonable request.
