# Supplementary material for: Phaeobacter inhibens induces apoptosis-like programmed cell death in calcifying Emiliania huxleyi
Source: Sci Rep. 2019 Mar 21;9:5215. doi: 10.1038/s41598-018-36847-6 (PMC6426857; doi:10.1038/s41598-018-36847-6)

Phaeobacter inhibens induces apoptosis-like programmed cell death in calcifying *Emiliania huxleyi*

Anna R. Bramucci and Rebecca J. Case

Supplemental Figure S1. ***Phaeobacter inhibens* induces nuclear blebbing in *Emiliania huxleyi*.** Epifluorescence microscopy of co-culture (8 d): overlaid with chlorophyll auto-fluorescence (red: emission 670-720 nm) and fluorescent DAPI stained DNA (blue: emission 417-477 nm). *E. huxleyi* cells where nucluear membrane appears to be blebbing are indicated by (B) and a nucleus, typical of a healthy cell, is indicated by (N). Scale bar is 5 µm.

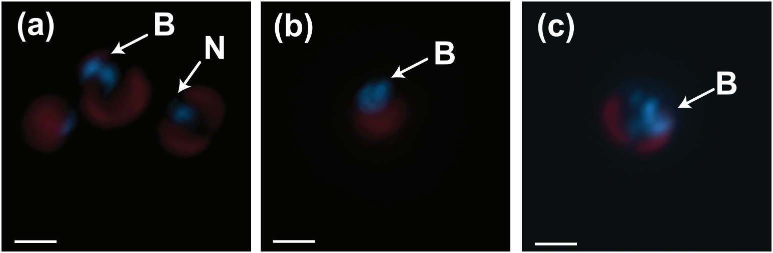

Supplemental Figure S2. Localization of caspase-like molecules within *Emiliania huxleyi* chloroplasts. Replicate *E. huxleyi* cells grown alone and in co-culture with *P. inhibens* are shown on 9 d. Each cell depicted as DIC (a,d,g), DIC overlaid with chlorophyll autofluorescence (red: excitation 610-650 nm; emission 670-720 nm) (b,e,h), and DIC overlaid with, fluorescence of active caspase-like proteases stained with specific pan-caspase marker: v-VAD-fmk (green: excitation 450-490 nm; emission 515-586 nm) (c,f,i). Scale bar is 5 μm.

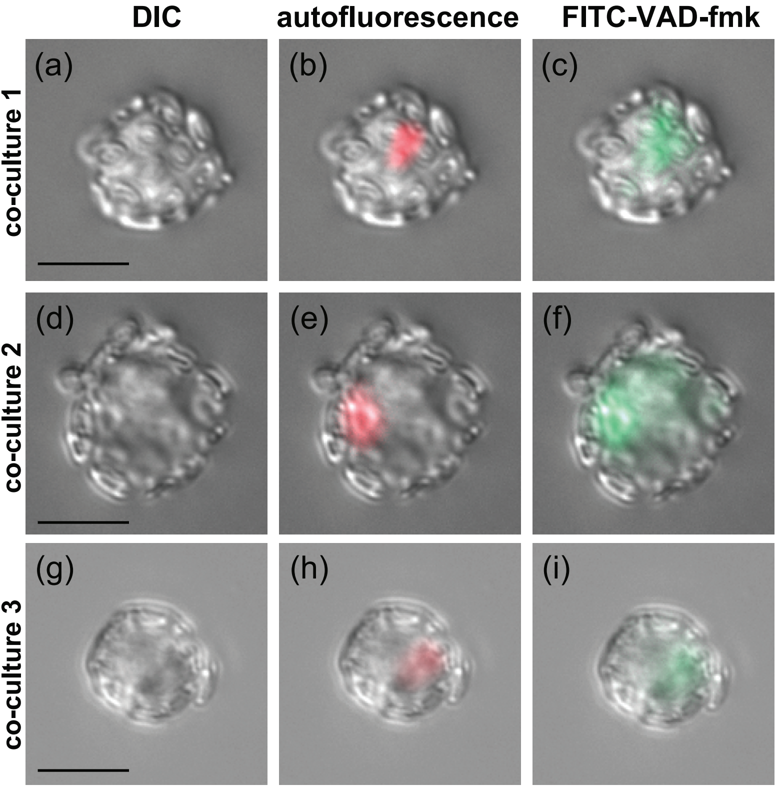

Supplement: Supplementary file 1 — Supplemental File [file 41598_2018_36847_MOESM1_ESM.pdf]
